# Newly Diagnosed Hemoglobin SC Disease Presenting With Choledocholithiasis With Acute Obstructive Cholangitis: A Case Report

**DOI:** 10.7759/cureus.84040

**Published:** 2025-05-13

**Authors:** Daniel T Tran, Justin J Youn, Gary Thompson

**Affiliations:** 1 Internal Medicine, Loma Linda University School of Medicine, Loma Linda, USA; 2 Ophthalmology, Loma Linda University School of Medicine, Loma Linda, USA; 3 Internal Medicine, Riverside University Health System Medical Center, Moreno Valley, USA

**Keywords:** acute cholangitis, choledocholithiasis, hemoglobin sc disease, hemoglobin ss disease, sickle cell disease

## Abstract

Hemoglobin SC disease (HbSC) is a sickle cell disease (SCD) that is considered to be mild compared to Hemoglobin SS (HbSS) disease. Both HbSS and HbSC can present with classic SCD complications such as hemolytic anemia, vaso-occlusive complications, functional aplenia, avascular necrosis, and cholelithiasis; however, HbSC generally has less severe and less frequent complications and can often be asymptomatic. As a result, HbSC is often underdiagnosed until a patient presents with a clinically significant complication. We present a case of newly diagnosed HbSC presenting with potentially life-threatening acute cholangitis due to choledocholithiasis. This case report serves to demonstrate that serious complications can occur in patients with HbSC. Additionally, early screening and diagnosis of HbSC in high-risk patients may allow for earlier treatment and potentially reduce the incidence of complications and increase patient quality of life.

## Introduction

Sickle cell disease (SCD) is a group of autosomal-recessive hemoglobinopathies resulting from different variations of point mutations occurring in the beta-globin gene. Hemoglobin SS (HbSS) is the most severe form of SCD and is caused by a point mutation of the codon for glutamic acid (GAG) to codon for valine (GTG), resulting in the replacement of glutamate with valine. In comparison, hemoglobin SC disease (HbSC) is a milder form of SCD where individuals inherit both the hemoglobin S (HbS) and hemoglobin C (HbC) genes. This occurs due to a point mutation of GAG to the codon for lysine (AAG), resulting in the replacement of glutamic acid for lysine [1].

In the United States, HbSS occurs in 1:941 births and HbSC occurs in l:6,173 births, making up approximately 30% of all patients with SCD [2]. The majority of clinical manifestations seen in SCD fall into the four major pathobiological processes, including HbS polymerization, vaso-occlusion, hemolysis-mediated endothelial dysfunction, and sterile inflammation [3]. Although the HbS trait or HbC trait does not individually lead to significant pathologic manifestations, HbSC has been noted to have similar clinical features as HbSS, but complications are often less frequent and less severe. Cholelithiasis, as seen in this case, remains one of the most common clinical manifestations of HbSC disease. A subset of patients with SCD will also have an increased risk of gallbladder disease and cholelithiasis, caused by polymorphic (AT) repeats found within the promoter region of the uridine diphosphate glucuronosyltransferase family 1 member A1 (UGT1A1) gene [4]. Other common clinical features seen in HbSC include hemolytic anemia, splenomegaly, aplastic anemia, vaso-occlusive complications such as renal papillary necrosis, osteonecrosis of bone, and proliferative retinopathy [5]. 

We present this case report demonstrating a newly diagnosed HbSC presenting with acute cholangitis due to choledocholithiasis. This case report serves to supplement the current literature about the associated complications seen in HbSC.

## Case presentation

A 35-year-old Hispanic American male with a significant medical history of choledocholithiasis, paternal family history of sickle cell trait, and no recent travel history presented to the emergency department with acute right upper quadrant abdominal pain and subjective fevers. He reported that 12 days prior to the current presentation, he was evaluated at another hospital’s emergency department with similar symptoms and was discharged with the recommendation to follow up with general surgery for an elective cholecystectomy.

On presentation, the vitals were as follows: Temp 99.8 °F (37.7 °C), BP 137/92, HR 108, RR 18, and O2 sat 98%. The physical examination was notable for diffuse abdominal pain without distension, rigidity, guarding, or rebound.

The laboratory data on presentation showed that the patient had a decreased hemoglobin (Hgb) with an elevated white blood count (WBC), platelets, alkaline phosphatase, aspartate aminotransferase (AST), alanine aminotransferase (ALT), and total bilirubin, as depicted in Table 1. 

**Table 1:** The trend of patient’s labs on admission, during admission, and at discharge. WBC: White blood cells, Hgb: Hemoglobin, AST: Aspartate transaminase, ALT: Alanine transaminase

Labs	On Admission	During Admission	At Discharge	Reference Ranges
WBC (/µL)	21.0	27.2	19.7	4.5 – 11
Hgb (g/dl)	11.5	10.3	11.0	13.5 - 17.5
Platelets (x10^9^/uL)	888	1,201	1,565	150 - 450
Reticulocyte Count (%)	-	4.85	-	0.5 - 2.5
Alkaline Phosphatase (U/L)	637	862	1040	30 - 130
AST (U/L)	82	140	109	8 - 48
ALT (U/L)	117	196	168	7 - 55
Total Bilirubin (mg/dl)	2.9	3.5	2.0	0.2 - 1.2

**Figure 1:**
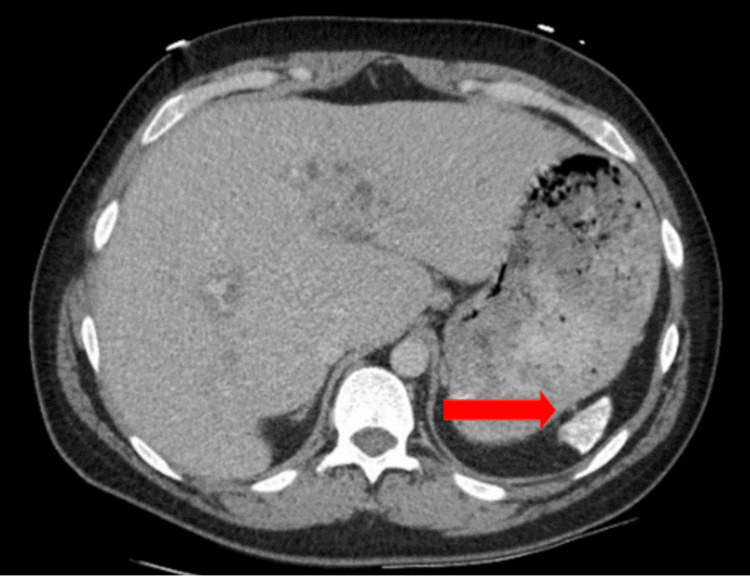
CT abdomen image of hypoplastic spleen. CT: Computed tomography

An abdominal ultrasound found cholelithiasis and sludge within the intrahepatic biliary tree and the gallbladder lumen. Computed tomography (CT) of the abdomen showed a dilated common bile duct (CBD) consistent with choledocholithiasis, bilateral hip findings suspicious for avascular necrosis, and hypoplastic spleen due to splenic infarcts (Figure 1).

The patient was treated with intravenous antibiotics for cholangitis. Endoscopic ultrasound (EUS) and endoscopic retrograde cholangiopancreatography (ERCP) demonstrated a dilated CBD and intrahepatic ducts with purulent bile and sludge, with subsequent successful balloon sweep and plastic biliary stent placement. General surgery evaluated the patient and performed an uncomplicated laparoscopic cholecystectomy.

The patient’s hospital course was complicated by recurrent fevers and persistent leukocytosis concerning persistent cholangitis. Repeat laboratory data showed a decrease in Hgb and an increase in WBC, platelets, alkaline phosphatase, aspartate aminotransferase (AST), alanine aminotransferase (ALT), and total bilirubin (Table 1).

**Figure 2:**
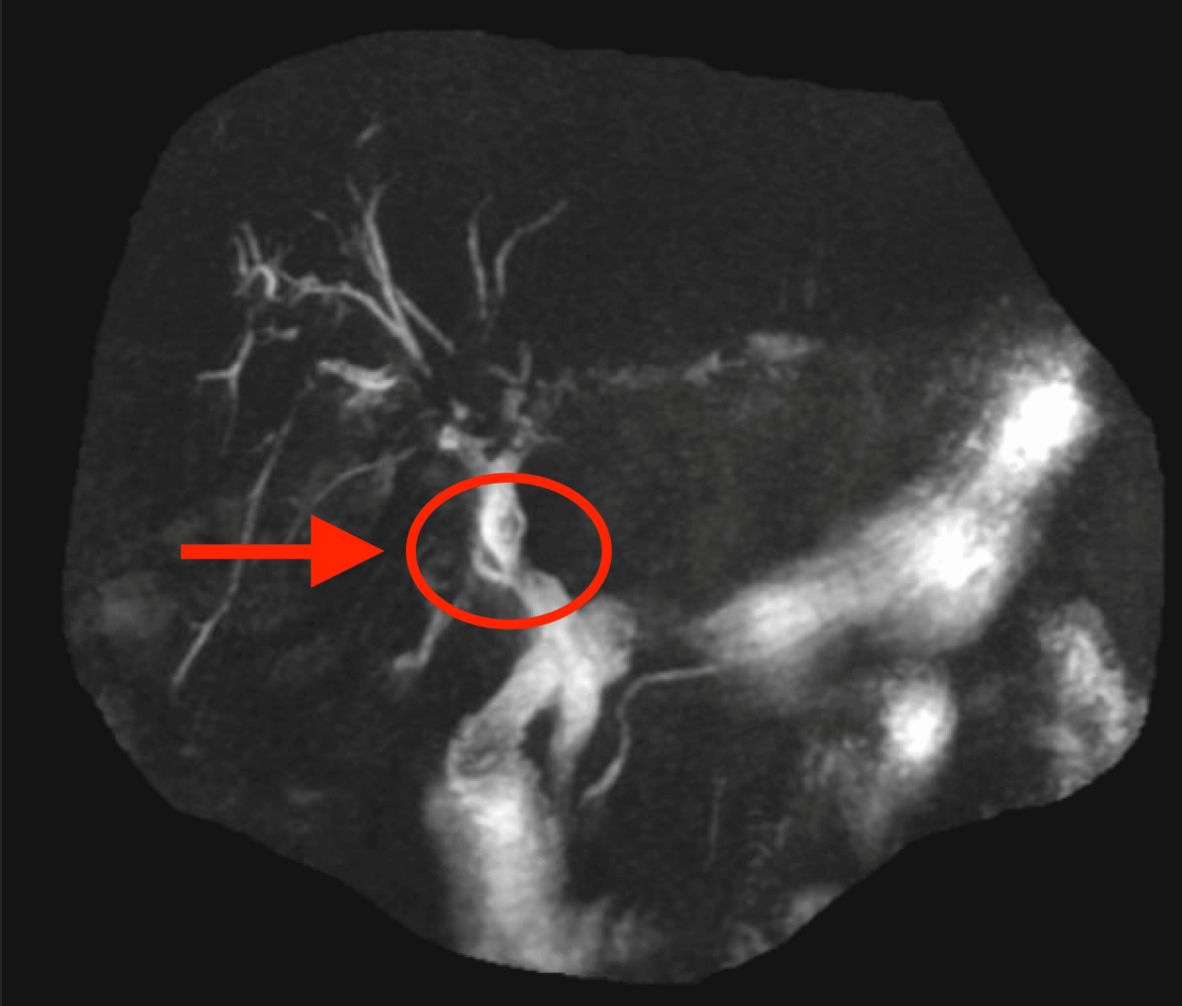
MRCP image of filling defect presumably representing a pigmented stone in the junction of the common hepatic duct and proximal CBD. MRCP: Magnetic resonance cholangiopancreatography, CBD: Common bile duct

A magnetic resonance cholangiopancreatography (MRCP) (Figure 2) showed a suggestion of a 5-6mm filling defect in the junction of the common hepatic duct and proximal CBD. Repeat ERCP was performed and showed an intact plastic stent and multiple filling defects in the right and left intrahepatic ducts (IHD). Balloon sweeps from bilateral IHDs were performed with extraction of several black pigmented stones. The original plastic stent was removed with placement of bilateral IHD double pigtail stents.

In the setting of a hypoplastic spleen, familial history of sickle cell trait, and acute choledocholithiasis at a young age, consideration of an underlying hemoglobinopathy was strongly considered. A hemoglobin electrophoresis was performed and showed findings consistent with hemoglobin SC disease as seen in Table 2.

**Table 2:** Results of patient’s electrophoresis during hospitalization showing findings consistent with hemoglobin SC disease.

Hemoglobin (Hb):	Results of Electrophoresis	Reference Ranges
Hb A	0.0%	96%-98%
Hb S	52.9%	0%
Hb A2	2.7%	<2.0%
Hb C	43.8%	0%

The patient’s condition improved through the hospitalization with a resolution of abdominal pain and fevers. Lab data upon discharge showed a continued increase in platelets and alkaline phosphatase, but improvements in all other lab values (Table 1). The patient’s persistent leukocytosis and thrombocytosis were attributed to the patient’s HbSC disease with functional asplenia and hyperactive bone marrow due to erythropoiesis stimulus.

A repeat elective ERCP for stent removal was planned for two weeks after discharge. He was discharged home with levofloxacin, metronidazole, folic acid supplementation, and follow-up with hematology for medical management, including consideration for hydroxyurea therapy for his newly diagnosed HbSC. The patient was also recommended to follow up with his primary care provider for recommended vaccinations, including pneumococcal, *Haemophilus influenzae* type b, and meningococcal vaccines.

## Discussion

HbSC is classically considered a mild form of SCD. The diagnosis of HbSC can often be delayed due to its indolent clinical course, with evidence that patients may not seek medical care until severe disease-related complications arise [2,6-7]. Current management recommendations call for deferring medical intervention unless the patient suffers a severe complication, which often occurs later in life compared to HbSS. Literature suggests that severe complications in HbSC patients typically occur after the age of 30 years [8].

Cholelithiasis is a relatively common feature of HbSS and other sickle cell-related hemoglobinopathies. In an analysis of 65 patients with hemoglobinopathies, gallstones were seen in HbSS in 11 of 42 patients (26%), while in HbSC, gallstones were seen in 3 of 15 patients (20%) [9]. In a larger cohort study of 96 Jamaican patients with HbSC disease, 18 of those patients (18%) were found to have gallstones [10]. A meta-analysis of adult SCD patients found a much higher prevalence (44.3%) of gallstones [11].

There is currently a lack of evidence-based management guidelines for patients with HbSC, as current management recommendations are inferred from studies with HbSS patients. Current literature suggests that patients diagnosed with HbSC are likely being under-treated in both low and high-resource settings, regardless of socioeconomic status and access to healthcare [11,12]. Currently, the first-line intervention for patients with HbSC is hydroxyurea (hydroxycarbamide) therapy, a ribonucleotide reductase inhibitor. However, hydroxyurea therapy is only indicated for patients with HbSC disease after patients have experienced at least one moderate to severe episode of sickle cell acute pain syndrome or acute chest syndrome within the last year. Intervention with hydroxyurea is also indicated if the patient experiences symptoms that heavily impact quality of life, including chronic pain, chronic kidney disease, priapism, chronic hypoxemia, anemia, and pulmonary hypertension.

While patients with HbSC typically do not manifest with comorbid complications as early in life as compared to HbSS, current literature reveals that early management and treatment of HbSC increases the quality of life and decreases the rate of pain crisis for patients with HbSC disease [12-14]. This highlights the importance of early screening for patients who are at high risk of developing HbSC disease.

Even though there are recommendations in place for universal screening for sickle cell trait, it was found that only 16% of patients who were positively screened and diagnosed at birth reported being aware of the diagnosis later in life [15]. This phenomenon is believed to be due to ineffective education for families that are diagnosed, and is believed to be more prevalent for patients with HbSC, as there is a much larger delay between diagnosis and symptomatic complications.

## Conclusions

This case report demonstrates a case where HbSC was not diagnosed until it manifested as a potentially life-threatening complication. Patients with undiagnosed sickle cell hemoglobinopathies in the setting of an acute complication often will require extensive diagnostic testing and workup to uncover the diagnosis. Performing a hemoglobin electrophoresis would allow ruling out other possible differential diagnoses of hemolytic anemia outside of SCD, such as hereditary spherocytosis, glucose-6-phosphate dehydrogenase (G6PD) deficiency, and autoimmune hemolytic anemia. To supplement the differential, it is also important to emphasize early screening methods through an extensive review of familial history and newborn screening results. As a result, this case report aims to suggest that earlier screening of patients and family members with known risk factors for HbSC may allow for more prompt monitoring and management of acute and chronic complications. This case also aims to suggest that patients with known HbSC disease may benefit from earlier intervention as well as an increased emphasis on patient education regarding the monitoring of their symptoms and close follow-up with their health care providers.
